# Nitric oxide and cytokinin cross-talk and their role in plant hypoxia response

**DOI:** 10.1080/15592324.2024.2329841

**Published:** 2024-03-24

**Authors:** Felix Lutter, Wolfram Brenner, Franziska Krajinski-Barth, Vajiheh Safavi-Rizi

**Affiliations:** a Institute of Biology, Department of General and Applied Botany, University of Leipzig, Leipzig, Germany; b Institute of Biology, Department of Plant physiology, University of Leipzig, Leipzig, Germany

**Keywords:** Nitric oxide, cytokinin, hypoxia, flooding tolerance, Nitric oxide and cytokinin interaction

## Abstract

Nitric oxide (NO) and cytokinins (CKs) are known for their crucial contributions to plant development, growth, senescence, and stress response. Despite the importance of both signals in stress responses, their interaction remains largely unexplored. The interplay between NO and CKs emerges as particularly significant not only regarding plant growth and development but also in addressing plant stress response, particularly in the context of extreme weather events leading to yield loss. In this review, we summarize NO and CKs metabolism and signaling. Additionally, we emphasize the crosstalk between NO and CKs, underscoring its potential impact on stress response, with a focus on hypoxia tolerance. Finally, we address the most urgent questions that demand answers and offer recommendations for future research endeavors.

## Nitric oxide role and biosynthesis

Nitric oxide (NO) is a small, but significant signal molecule in plants.^1–4^ It is crucial to many biological plant processes at each step of the plant life cycle.^5^ For example, NO is involved in plant growth, development, and metabolism.^1–5^ Furthermore, it mediates responses to both abiotic and biotic stresses, including responses to hypoxia, salt stress, and drought to name only a few potential abiotic stresses, as well as plant-pathogen interaction as a potential source of biotic stress.^1–5^ In particular, it has been documented that NO plays a pivotal role under hypoxic conditions, influencing the regulation of cytochrome-c-oxidase (CytOX) and alternative oxidase (AOX) activities. This regulatory role extends to the modulation of mitochondrial oxygen consumption.^6^

**Figure 1.**
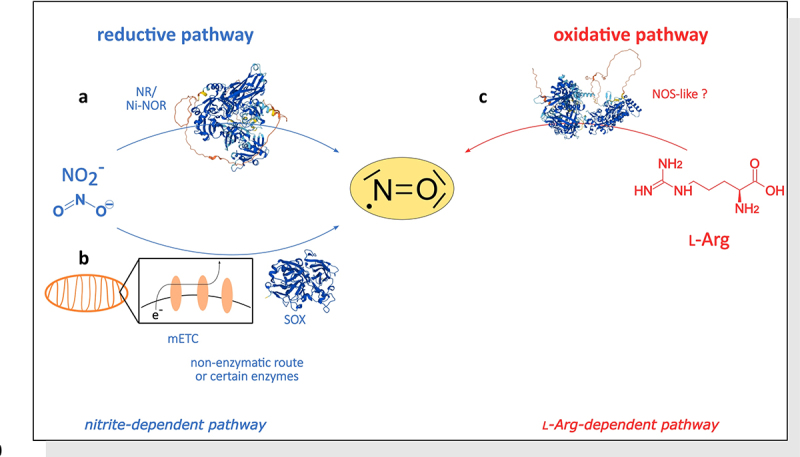
Biosynthetic pathways of NO. The biosynthesis is classified in a reductive (a, b) and in an oxidative (c) pathway. In the nitrite-dependent pathway, nitrate (NO_3_^−^) is first reduced by the cytosolic NR, releasing nitrite (NO_2_^−^). Nitrite is then reduced again by (a) the cytosolic NR or by root membrane-bound Ni-NOR (structure: NIA1 from *Arabidopsis thaliana*), producing NO. Mohn et al. (2019) demonstrated that the NIA1 isoform is the more efficient nitrite reductase and therefore this isoform produces mainly NO.^7^ (b) Additionally, nitrite can be reduced by the mitochondrial electron transport chain (mETC) under hypoxia, and under certain conditions by other enzymes such as sulfite oxidase (SOX, structure: SOX from *Arabidopsis thaliana*) or aldehyde oxidoreductase. A non-enzymatic source of NO is the reaction of NO_2_ and carotenoids in light. (c) The oxidative pathway includes an l-Arg-depending reaction, which is catalyzed by a NOS-like enzyme (structure: NOS2 from *Homo sapiens*) in plants. This figure is modified after Besson-Bard, Pugin and Wendehenne (2008). 3D-structure of the proteins are obtained from AlphaFold.^1,3,8–11^.

NO can be produced through either oxidative or reductive pathways, and both pathways are achievable through enzymatic or nonenzymatic processes (see Figure 1).^2,3^ Two enzymatic pathways are recognized for NO production: a nitrate/nitrite-dependent pathway and an l-Arginine-dependent pathway.^1–3,8^ The first pathway includes the cytosolic nitrate reductase (NR), which primarily reduces nitrate to nitrite using NAD(P)H as electron donor, but NR can also reduce nitrite further to NO.^2–5^ A similar reaction is found in roots, where a root-specific membrane-bound nitrite-NO reductase (Ni-NOR) catalyzes the reduction of nitrite to NO. This reaction is coupled with a membrane-bound NR, that produces nitrite from nitrate.^2–4^ *Arabidopsis* has two known NR genes: *NIA1* and *NIA2*.^5^ Different studies show that the double mutants of *nia1 nia2* had a reduced NO level, while the *NIA1 NIA2* overexpression line showed an elevated NO level under normoxia (i e., the oxygen of about 21% O_2_ at standard atmospheric conditions).^5,12,13^ The latter result suggests that the NR-dependent pathway has the major role in NO production under normoxia.^12^ Additionally, Mohn et al. (2019) demonstrated that the *NIA1* isoform of the NR is a more potent nitrite reductase than the *NIA2* isoform.^7^ Therefore, *NIA1* produces mainly NO, while *NIA2* is mainly involved in the nitrate reduction.^7^

The second pathway, which uses l-Arginine as substrate, produces NO and l-Citrulline using a nitric oxide synthase (NOS) as the catalyzing enzyme. Although inhibitors of mammalian NOS have successfully suppressed NO synthesis in higher plants, the plant NOS homolog has not yet been identified.^1–3,5^ The only NOS homolog identified in plants was found in a green alga (*Ostreococcus tauri*), to which no homolog has been found in higher plants.^2,5^ This suggests that there is a NOS-like enzyme in higher plants that has not yet been described.

Other potential pathways that may produce NO in plants are the reduction of nitrite to NO using electrons from the mitochondrial electron transport chain during hypoxia.^8,9,14^ Furthermore, sulfite oxidase, xanthine oxidoreductase, and aldehyde oxidoreductase may produce NO under certain conditions.^1,9,12^

Nonenzymatic sources of NO are the uptake from the atmosphere, as a product of the reaction between NO_2_ and carotenoids in light, and as a result of the conversion of nitrite to NO under acidic conditions in response to abscisic acid and gibberellins.^3,5,9^ The conversion of nitrite to NO in acidic condition (equation 1), like in the apoplast, occurs spontaneously and is slow.^15,16^

(1) $$2N{O_{2}}^{-}+2H^{+}⇌2\mathrm{HN}O_{2}⇌\mathrm{NO}+NO_{2}+H_{2}O⇌2\mathrm{NO}+0,5O_{2}+H_{2}O$$

Due to its gaseous nature, its small size and its ability to cross membranes without any carrier, NO can diffuse both within and between the cells.^5^

NO signaling manifests through both transcriptional and post-translational responses.^2–5^ Transcriptional responses primarily involve stress-related genes,^4^ which play diverse roles ranging from plant defense and oxidative stress responses to hypoxic responses and developmental processes.^4^ Notably, endogenous NO levels serve as crucial signals for gene regulation under hypoxia and even preceding hypoxia onset.^2,17,18^

Hartman et al. (2019) demonstrated that NO depletion, facilitated by ethylene, results in the accumulation of the ERFVII transcription factors (Group VII of ethylene response factor). This accumulation aids plants in pre-adapting to impending hypoxia.^17^

ERFVIIs are able to sense O_2_ and NO through the Cys-branch of the N-degron pathway and are involved in regulation of hypoxia-adaptive genes^17–20^ It is shown that ethylene induces the expression of *PHYTOGLOBIN 1* (*PGB1*), a potent NO-scavenger, resulting in the depletion of NO.^17^ During hypoxia, a decreased NO level mediated by ethylene leads to the stabilization of ERFVII and induction of hypoxia-responsive genes.^18^

NO is involved in seed germination, hypocotyl growth, floral transition, adventitious root formation, and senescence.^21–26^ Belgini and Lamattina (2000) reported that NO induces seed germination.^21^ After 48 h of imbibition in 100 µM SNP (sodium nitroprusside) almost all seeds of lettuce (*Lactuca sativa* l. cv. Grand Rapids) break dormancy and germinated in darkness at 26 °C.^21^ In contrast, lettuce seed imbibed with water or nitrate/nitrite didn’t break dormancy.^21^ Adding the NO-scavenger cPTIO (2-(4-carboxyphenyl)-4,4,5,5-tetramethylimidazoline-1-oxyl-3 oxide) to the SNP solution completely abolished the dormancy break.^21^ Lettuce seeds exhibit a thermoinhibition of germination in darkness above 25 °C.^21^ These results are in line with findings in other plant species, such as *Lycopersicon esculentum*, *Arabidopsis*, *Hordeum vulgare*, and *Paulonia tomentosa*, concluding that NO plays a crucial role in germination.^24^ In addition, an upregulation of abscisic acid 8’-hydroxylase was reported when NO was released from SNP, resulting in higher catabolism of ABA and therefore promoting seed germination in *Arabidopsis*.^25^

Nitric oxide (NO) plays a significant role in the flowering process of the *gsnor1–3* mutant line.^27^ In *Arabidopsis*, the *gsnor1–3* line, characterized by loss of function in *S*-Nitrosoglutathione reductase (GSNOR) and consequent accumulation of *S*-nitrosylated compounds, exhibits reduced *FLOWERING LOCUS C* (*FLC*) expression. This reduction in *FLC* expression is associated with promoting flowering in the *gsnor1–3* mutant.^27^

## NO-mediated posttranslational modifications (PTMs)

Post-translational protein modifications (PTMs) by NO appear as reversible nitrosylation of cysteine residues, nitration of tyrosine residues, and nitrosylation of metals.^3,5,8^ Additionally, NO can also modulate Ca^2+^ signaling.^3,5,8^ The most prevalent PTM by NO is the *S*-nitrosylation of Cys-residues, in which the thiol group of Cys reacts with NO.^3,5,8^ One possible mechanism of this reaction is proposed to be an electrophilic attack of the nitrosonium cation (NO^+^, product from NO oxidation) on thiolate, but there are more potential mechanisms to form *S*-nitrosothiols.^3^ *S*-nitrosylation of Cys-residues can either promote or inhibit the formation of disulfide bonds within neighboring thiols, thereby possibly modulating the secondary structure of proteins.^3^ *S-*nitrosylation modulates protein function by regulating enzyme activity, protein localization, the ability to interact with other proteins, protein degradation, and protein-DNA binding.^12^ Although there are many potential target proteins for *S-*nitrosylation (about a thousand in GSNO-exposed leaf protein extract, and about 50 identified proteins in *Arabidopsis thaliana* in absence of any NO donor), there is no primary consensus sequence identified so far determining the potential of *S*-nitrosylation of Cys-residues.^8,28^ *S*-nitrosylated Cys-residues are redox modifications and are reversible PTMs.^8^ Furthermore, *S*-nitrosylation of Cys_10_ of *S*-nitrosoglutathione reductase (GSNOR) leads to conformational changes, resulting in a selective autophagy degradation of GSNOR.^29^ This NO-mediated degradation is positively regulating hypoxia responses, linking NO signaling and autophagy during hypoxia.^29^

Several studies have provided detailed analyses of *S*-nitrosylated proteins, including investigations into RuBisCO, glyceraldehyde-3-phosphate dehydrogenase (GAPDH), and salicylic acid binding protein 3 (SABP3).^4,12,30^ SABP3 plays a crucial role in plant defense responses, with research demonstrating that pathogen-induced nitrosative bursts mediate the *S*-nitrosylation of Cys_280_ of SABP3. This modification suppresses the binding of salicylic acid to SABP3, thereby reducing the carbonic anhydrase activity of the enzyme.^4,12,30^ Despite its importance in plant defense, *S*-nitrosylation may establish a negative feedback loop that modulates plant defense responses.^4,12,30^

Another NO-mediated PTM is tyrosine nitration which occurs in a reaction between peroxynitrite (ONOO^−^) and tyrosine residues of proteins. Peroxynitrite is formed in the reaction of NO with a superoxide anion (O_2_^●^) and is considered to be a powerful nitration reagent.^4,8,31^ According to this fact, a high amount of peroxynitrite is toxic and leads to lipid nitration as well as DNA and protein damage.^8,32^ The result of the reaction of peroxynitrite and tyrosine residues is 3-nitrotyrosine, which, depending on the protein, can activate or inhibit its function due to conformational changes.^3,4,8,32^

The conformational changes of tyrosine nitration are caused by the addition of a nitro group to the aromatic ring system of Tyr resulting in a decrease of its pK_a_ (acidity constant) of about 3 pH units.^4^ Tyrosine nitration might also act as a mediator of NO signaling during plant defense responses.^31,32^ The accumulation of peroxynitrite in pathogen infection and the related increase of Tyr-nitrated proteins suggests that this PTM may mediate NO signaling during the hypersensitive response.^32^ In general, Tyr nitration is considered a nitrosative stress marker.^31,32^ Furthermore, Tyr nitration appears to be involved in the modulation of many metabolic pathways.^12^ So far there is no known pathway for the reversal of Tyr nitration, suggesting that tyrosine nitration is probably an irreversible PTM.^3,8,12,32^

Metal nitrosylation, another NO-mediated PTM, occurs by coordination chemistry, where NO is coordinating the central metal ion in metal-containing proteins.^3,8,33^ Metal nitrosylation can lead to the inhibition of protein activity.^3^ Putative targets for metal nitrosylation are for example symbiotic leghemoglobin or nonsymbiotic PGB1 or PGB2, called phytoglobins (PGBs).^3,8,34,35^

## Cellular NO homeostasis

NO and its derivatives such as peroxynitrite are potent oxidants and their accumulations are associated with detrimental effects on the cells.^32^ Therefore, the cellular NO level needs to be tightly regulated to ensure an optimal balance in NO signaling.^8,31,32^ For maintaining NO homeostasis, plants employ glutathione (GSH) and PGBs as NO scavengers.^8,31,32^ GSH, featuring a Cys residue with a thiol group, serves as a crucial component susceptible to *S*-nitrosylation at the thiol group, leading to the formation of *S*-nitrosoglutathione (GSNO), a low molecular weight *S*-nitrosylated compound.^3,4,8,12,30,36^ GSNO, a major NO reservoir, can be dissociated by various means.^3^ NO can be released non-enzymatically from GSNO by light (photolytic), in redox reactions, or by hydrolysis of GSNO.^36,37^ Moreover, GSNO is able to transfer NO directly to other putative protein targets in a transnitrosylation reaction. Transnitrosylation is catalyzed by transnitrosylase enzymes, which carry and transfer the NO group to the target molecules.^12,30,32,36^ GSNO itself is recognized to exhibit transnitrosylation activity in plants, operating independently of enzymatic involvement.^30^ Consequently, GSNO affects the nitrosylation degree of proteins and peptides, and modulates the cellular *S*-nitrosothiol content.^8,30,32,36^

GSNO reductase (GSNOR) is a key enzyme for regulating cellular *S*-nitrosothiols and catalyzes the reaction of GSNO to glutathione disulfide (GSSG) and ammonia using NADH as a co-substrate.^3,8,12,14,30,36–38^ GSNOR is an evolutionarily conserved enzyme found from bacteria to mammals and plants, playing a crucial role in cellular function.^3,36^ Loss-of-function mutants of GSNOR do not only accumulate GSNO and consequently have higher NO levels, but also contain higher levels of nitrate and nitroso species.^4,36^ The activity of GSNOR could be regulated by *S*-nitrosylation through GSNO or NO.^5,36^ This suggests that GSNOR is establishing a fine-tuned feedback loop maintaining the NO homeostasis in the cell through *S-*nitrosylation of the NO-quenching GSNOR enzyme.^36^

The second class of key compounds for NO homeostasis are phytoglobins (PGBs), which are considered to be major NO scavengers.^8,34^ PGBs contain a central Fe^2+^ ion that is coordinated to four nitrogen atoms, belonging to the four pyrrole rings of the heme group, and two histidine (proximal and distal) residues of the protein.^34,35^ The distal histidine ligand can be substituted e.g. by oxygen or NO.^34,35^

There are three important sub-classes of non-symbiotic PGBs, which are class 1, class 2, and class 3.^33,34^ Class 1 PGBs have a very high affinity for oxygen.^34^ This is an optimal condition for the oxygen-dependent NO scavenging mechanism.^34^ The class 2 of non-symbiotic PGBs is characterized by a lower oxygen affinity caused by tighter hexacoordination.^34^ This condition results in a less efficient NO scavenging but enables the class 2 PGBs to sense low oxygen levels and store oxygen.^34^ Class 3 or truncated PGBs have a low oxygen affinity and might be involved in NO metabolism in some green algae and dicots.^12,33,35^

The scavenging mechanism of PGBs (non-symbiotic class 1) is described by their NO dioxygenase (NOD) activity, a reaction in which oxidation of NO to nitrate occurs using NAD(P)H as an electron donor.^33–35,39^ This reaction requires oxygenated PGBs (PGB-O_2_), which are containing Fe^2+^ ions.^8,33,35^ During the oxidation of NO to nitrate, the Fe^2+^ ion is also oxidized to Fe^3+^, which results in an oxidized PGB (metphytoglobin).^33–35^ Metphytoglobin is then reduced, using NAD(P)H as an electron donor, and subsequently associates with oxygen to form PGB-O_2_.^33,35,39^ Nevertheless, a dedicated reductase for metphytoglobin has not been identified thus far.^35^ Possible candidates for serving as reductases for metphytoglobin include free flavins. These compounds may function as electron carriers from NADH, working in conjunction with cytosolic NR and the flavoprotein ferredoxin NADP^+^ oxidoreductase.^35^ Additionally, deoxyferrous PGBs can tightly bind NO resulting in precluding oxygen transport.^33^ According to the study by Rubio et al. (2019), there is some evidence that the scavenging function of PGBs via NOD activity is not through *S*-nitrosylation within the PGBs themselves, but rather related to their heme groups.^33^

PGBs link the cytosolic NR with the mitochondria. The NR reduces nitrate to nitrite, which is transported to the mitochondria.^8,9,12,14^ Here, nitrite is reduced to NO at different sites (complexes III and IV, and possibly by AOX), and diffuses to the cytosol, where it is scavenged by PGBs to produce nitrate.^8,9,12,14,34^ The nitrate is released to the cytosol, where it can be utilized by the cytosolic NR.^9,14,34^ This cycle is restricted to tissues exposed to hypoxia, as oxygen can readily inhibit the reactions inside the mitochondria.^8,9^ During hypoxia, this cycle may help to maintain the redox and energy status through aiding fermentation by oxidizing NAD(P)H.^33,35^

As another means of keeping NO homeostasis, there is evidence suggesting that the phytohormone cytokinin may function as an NO scavenger in plant cells.^40^ This finding supports a potential interaction between NO and cytokinin, a topic that will be further elucidated in subsequent sections of this review.

## Cytokinin

Cytokinins (CKs) are important plant hormones that influence plant development and growth and mediate responses to biotic and abiotic stresses. For example, they influence shoot and root size, shoot and root meristem activity, leaf senescence, and chloroplast development. Furthermore, they are involved in biotic and abiotic stress reactions, such as pathogen defense, drought stress, or high or low temperatures.^41–46^ Cytokinins act antagonistically to auxins and thus also determine key developmental events as well as the habitus of the plant. As an example, both hormones act antagonistically on the specification of the primary root during embryo development and the formation of lateral roots.^41,43,47^ Cytokinins also interact with most other hormones. Generally, the role of each plant hormone should not be considered in isolation.^41^

Chemically, cytokinins are adenine derivatives, substituted at the *N*^*6*^ atom with an isoprenoid or aromatic side chain, according to which cytokinins are classified.^41,43,48^ Isoprenoid CKs are the most common class of CKs.^49^

**Figure 2.**
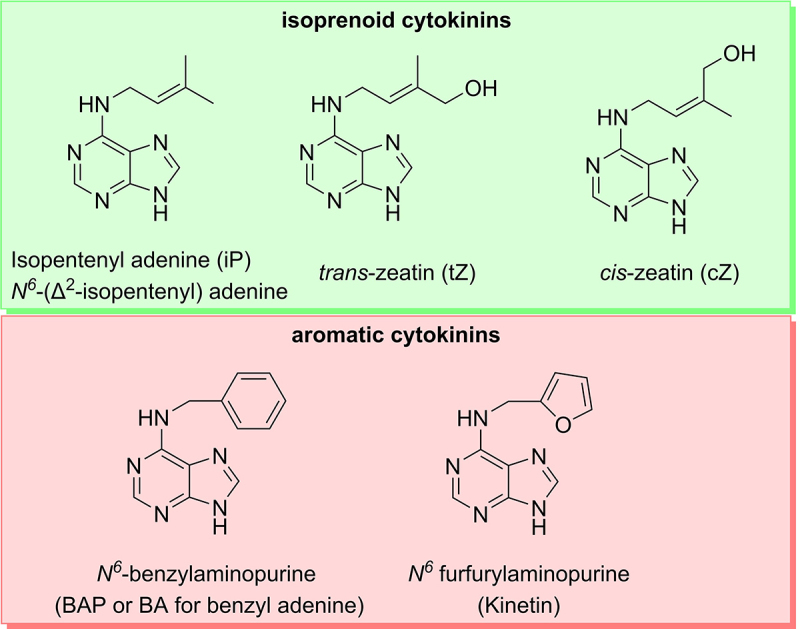
Molecular structure of exemplary representatives of cytokinins. Cytokinins are classified by their side chain as isoprenoid and aromatic cytokinins. Isoprenoid cytokinins (shown in the green box) are represented by isopentenyl adenine, dihydro-zeatin, *trans*-zeatin and *cis*-zeatin, while the examples for aromatic cytokinins (red box) are benzyl adenine and kinetin. ^43,48.^

Naturally occurring CKs in *Arabidopsis* are *trans*-zeatin (tZ), isopentenyl adenine (iP), and dihydrozeatin (dhZ) and their derivatives, which all can be assigned to the isoprenoid CKs (see Figure 2).^48^ *Cis-*zeatin (cZ) represents another naturally occurring isoprenoid CK, which is found ubiquitously but is biologically active only in some plant species.^41,43,48^ Additionally, dihydrozeatin may be synthesized through the reduction of the double bond in the side chain of tZ catalyzed by a zeatin reductase, but a recent investigation suggests that this pathway needs to be investigated further due to the low basal activity of the zeatin reductase.^41,49^ The isoprenoid CKs named above are contrasted by the aromatic CKs, which occur naturally only in some species and are mostly used as synthetic compounds.^43,48^ Furthermore, it should be mentioned that all natural CKs occur not only as free bases but also as the corresponding nucleosides, nucleotides, and glycosides.^43,48^ While the free bases of CKs are the physiologically active compounds, their nucleoside, nucleotide and glycoside conjugates are physiologically less active or inactive compounds.^43,48,50–52^

The biological and physiological activity of the naturally occurring CKs varies depending on their molecular structure and species. *Trans*-zeatin has a higher biological activity than *cis*-zeatin and is the active CK form in most if not all land plant species.^42,43,48,52^ The differential activity of compounds with cytokinin activity is reflected in their differing binding properties to the three receptors (see below).^53^

## Cytokinin metabolism

Cytokinins are synthesized via two different metabolic pathways. In the first pathway, cZ is produced by the degradation of tRNA using a specific adenylate isopentenyl transferase (tRNA-IPT).^41,42,45,48,49^ This pathway is of minor importance in *Arabidopsis* and probably most other plants since it can not produce sufficient amounts of CKs because the rate of tRNA metabolism is too low.^41,43,48^ The second and more important pathway is a *de novo* synthesis of CKs catalyzed by adenosine-dependent isopentenyl transferases (IPT). After further reaction steps, either tZ or iP can be formed.^41,43,48,49,51^ The isopentenyl transferase uses adenosine in the form of AMP, ADP, or ATP as a substrate and substitutes an H atom for an isoprenoid side chain at the *N*^*6*^ atom of adenine.^41,43,48^ The isoprenoids involved in this reaction are dimethylallyl pyrophosphate (DMAPP) and hydroxymethylbutenyl diphosphate (HMBDP), taken either from the mevalonate pathway (MVA pathway) or from the methyl erythritol phosphate pathway (MEP pathway).^43,48^ HMBDP is formed as an intermediate in the MEP pathway, which is localized in the plastids of higher plants.^48,54^ In contrast, DMAPP is an intermediate of both the MEP pathway and the MVA pathway, with the latter pathway being localized in the cytosol.^48,54^ The intermediates formed by isopentenyl transferases are the compounds isopentenyladenosine-5’-triphosphate (iPRTP) and isopentenyladenosine-5’-diphosphate (iPRDP), which become dephosphorylated to isopentenyladenosine-5’-monophosphate (iPRMP).^49^ iPRMP represents a precursor for a broad spectrum of other CK metabolites.^49^ Whereas iP-type CKs are formed directly from iPRMP, synthesis of tZ-type CKs additionally requires the hydroxylation of the isoprenoid side chain by a cytochrome P450 enzyme.^43,49,51,55^ If HMBDP is used as a substrate in the reaction, tZ is formed. iP is produced when DMAPP reacts with adenosine.^48^

The most active forms of CKs are the free bases.^43,45,48,51^ They can be synthesized from their nucleotide precursors by cleavage of the ribose from the adenine. This step is catalyzed by enzymes of the LONELY GUY (LOG) family.^43,45,51,56^

The amount of active CKs in plant tissues is reduced in two ways, either by reversible or irreversible conjugation with sugars or by cleavage of the adenine from the side chain.^41,43,45,48,49,51,57^ The sugar conjugates are less active or inactive.^43,48,52^ Glycosylation can occur at the hydroxyl group of the isoprenoid side chain (*O*-glycosylation) or the *N*^*3*^, *N*^*7*^, and *N*^*9*^ positions (*N*-glycosylation) of the purine ring.^48,58^ *O*-glycosylation is a reversible reaction catalyzed by *β*-glycosidases.^43,48,49,59^ *N*-glycosylation was thought to be an irreversible modification of CKs resulting in their permanent deactivation, but recent results suggest that *N*-glycosylation might be a reversible modification.^49^ In addition, the *O*-glycosylated conjugates of CKs represent a stable storage form of CKs.^48^ Oxidative cleavage of the side chain from the adenine, which is catalyzed by cytokinin oxidase/dehydrogenases (CKX) in one enzymatic step using molecular oxygen as an oxidant is an irreversible reaction releasing free adenine or adenine nucleosides and the aldehydes of the side chain.^41,45,48,51,57^ The CKX enzymes can use all isoprenoid CKs, such as iP or tZ and their ribosyl derivatives, by recognizing the double bond in the side chain, whereas the aromatic CKs are more or less resistant to degradation by CKX enzymes.^41,43,48,49,60^ Interestingly, it has been reported in *Arabidopsis* that in addition to the free bases, iP *N*^*9*^-glucoside is a substrate for AtCKX1, AtCKX2 and AtCKX7, whereas iP *N*^*9*^-, tZ *N*^*9*^- and cZ *N*^*9*^-glucosides are substrates for CKX enzymes in maize.^49^ The *Arabidopsis* genome contains seven CKX genes, named *AtCKX1* to *AtCKX7*.^49,61^ Their organ- and tissue-specific and subcellular localizations differ according to their functional specification, thus enabling strictly localized manipulation of the CK status (see Table 1).^61^

**Table 1.** CKX enzymes and their extra- and intracellular localization.^61–63^

Gene	Expression in tissue or organ	Subcellular localization
*AtCKX1*	Hypocotyl, radicle, stem, shoot apex	Vacuole
*AtCKX2*	Stamen	ER lumen, extracellular space
*AtCKX3*	Callus, cell suspension, sepal	ER, vacuole
*AtCKX4*	Adult rosette leaves	Extracellular space
*AtCKX5*	Callus, stamen, sepal, senescing leaves	Extracellular space
*AtCKX6*	Hypocotyl, shoot apex, nodes	Extracellular space
*AtCKX7*	Vascular tissue of seedlings, female gametophyte	Cytosol

Due to their strong effects on plant physiology and development, the amount of active CKs is strictly regulated in spatio-temporal patterns. This is accomplished by fine-tuning of the expression of *IPT* and *LOG* genes in co-ordination with the different cytokinin deactivation pathways outlined above in order to establish a suitable cytokinin level for each tissue, developmental stage and environmental influence.^46,64^ Undirected overexpression of *IPT* genes leads to abnormally high endogenous CK levels resulting in inhibition of a normal root system formation and severe morphological and physiological abnormalities in the shoot.^46^ To achieve a beneficial effect on plant development, only a minimal increase in the *IPT* expression is sufficient.^46^ Localized control of the amount of active CKs to benefit yield can also be achieved by reducing the activity of specific *CKX* genes.^65,66^ Beneficial effects of an increased CK level may include better maintenance of photosynthetic rate, higher nitrate flux, enhanced drought tolerance, and delayed senescence resulting in improved plant growth, increased biomass, higher shoot apical meristem activity, and enhanced seed yield.^46,65–67^ Furthermore, cold tolerance and salt tolerance may also be increased.^64^

## Cytokinin transport

Cytokinins are considered mobile signals, for which their intra- and intercellular mobility and differential translocation of different types of CKs are crucial to their differential function.^51^ In general, root-synthesized tZ-derived types of CKs are transported acropetally in the xylem, while shoot-synthesized iP-types of CKs are carried basipetally or systemically through the phloem.^51,68–70^ Several different transporters have been identified whose function is associated with the translocation of CKs.^43,51,68,69,71,72^ The first transporter identified to be a CK transporter was ABCG14, a plasma membrane-localized CK exporter belonging to the ATP-BINDING CASSETTE TRANSPORTER SUBFAMILY G (ABCG).^51,68,69,71,73,74^ This transporter is involved in root-to-shoot transport of tZ-type CKs through the xylem. It is expressed in the pericycle and phloem companion cells of the *Arabidopsis* root exporting tZ into the apoplast.^51,68,69,71,73,74^ Mutants of *ABCG14* show shoot growth retardation, smaller inflorescences, smaller rosettes, slender stems with a reduced number of vascular bundles, and low levels of CK in the shoot.^51,68,69,73,74^

The second class of CK transporters belongs to proteins of the PURINE PERMEASE (PUP) family.^51,68,69,75,76^ As an example, PUP14 is localized to the plasma membrane importing bioactive CKs (free bases or nucleobases, respectively) into the cytoplasm.^51,68,69^ It negatively regulates the apoplastic CK pool, and its expression pattern in the shoot apical meristem, root, and embryo is inversely correlated with the CK response.^51,69^ In loss-of-function mutants of *PUP14*, an upregulation of CK signaling was observed, suggesting that spatiotemporal *PUP14* expression is involved in the regulation of the local CK status.^68,69^ A model in which PUP14 functions by depleting apoplastic CK through its transport into the cell resulting in degradation is debated.^77^

Furthermore, members of the EQUILIBRATIVE NUCLEOSIDE TRANSPORTER (ENT) family, which are known to transport purine and pyrimidine nucleosides, may also act as influx carriers for CKs.^51,68,71,78,79^ Although an *ent1* mutation does not affect the CK response substantially, the *ent3* mutant shows a reduced endogenous level of CKs in the roots. Surprisingly, this mutant also shows an enhanced capacity to deliver externally supplied tZ riboside to the shoot, which indicates that the ENT3 transporter might be involved in the CK distribution in plants.^51,78,79^ While the role of ENT3 as a CK transporter is still somewhat unclear, a screen for suppressor mutations of *IPT* overexpression suggests that AtENT8 acts as a potential CK transporter.^71^

Additionally, transporters belonging to the ABC subfamily I (ABCI) and AZA-GUANINE RESISTANT (AZG) are implicated in CK transport.^51,71,72^ The latter family was originally identified as adenine and guanine importers, which also import CKs.^51,80^ An enhanced ability to accumulate adenine was observed in an overexpression line of *AZG1* in *Arabidopsis*, while *azg1* mutants show a reduced sensitivity to exogenously applied CKs. Furthermore, AZG2 is localized to the ER and the plasma membrane in root primordia and modifies the distribution of CKs, inhibiting lateral root formation.^51^ The *Arabidopsis* genome contains two genes encoding AZG transporters.^71^ It was reported that AtAZG1 may function as an active transporter of CKs, while AtAZG2 may act as a diffusion facilitator.^71^ Recently, the structure of AZG1 was determined by the work of Xu et al. (2024), and a transport model was proposed.^72^ AZG1 forms a homodimer containing a transmembrane and transport domain, allowing the import of CKs using the electrochemical potential of protons in a pH dependent manner.^72^

In summary, the issue of cytokinin transport and the generation of local maxima and minima of cytokinin action appears to be far from solved. Most candidate transporters appear to have a broad substrate spectrum and act too nonspecifically to answer many questions of cytokinin distribution.

## Cytokinin signal transduction

CK signaling in plants is mediated by a multistep His-Asp phosphorelay system specific to plants and derived from the bacterial two-component system. In this system, a phosphoryl group is sequentially transferred from the CK receptors to the downstream components detailed below, alternating between His and Asp residues.^41,43,71,81^ In plants, CKs are perceived by binding to the extracytoplasmatic CHASE domain of the histidine kinase receptors (CHK) located mostly in the ER membrane and to a lesser extent in the plasma membrane.^41,43,71,82,83^ It should be mentioned that at this point both sites of perception are thought to be crucial for CK signaling, but the functional differentiation is still unclear.^51,84,85^ Binding of CKs to CHK triggers the autophosphorylation of a conserved His residue. Subsequently, the phosphate is transferred to a conserved Asp residue within the receiver domain of the receptors. The phosphate is then transferred to a conserved His residue of the phosphotransfer proteins (HPT), which shuttle between the cytoplasm and the nucleus. In the nucleus, the phosphate is transferred to conserved Asp residues of the type-A and type-B response regulators (RRA and RRB, respectively). The type-B response regulators are active transcription factors in the phosphorylated state, activating the transcription of their target genes.^41,43,44,71^ A number of these target genes encode type-A response regulators, which inhibit the upstream CK signaling in a negative feedback loop. The exact mechanism of their negative feedback activity is not yet clear and needs to be investigated.^43,71^

Furthermore, type-C response regulators (RRC) have been identified, as a distinctive group after originally having been assigned to the RRAs.^71^ Type-C RRs are structurally similar to type-A response regulators, but their primary structure is more similar to the receptor receiver domain.^71^ CKs do not upregulate their expression and the expression is spatially restricted to reproductive organs.^71^ At this point, it is important to note that NO negatively regulates CK signaling.^43,44,71^

## Confirmed NO-CKs interactions and implications for plant stress tolerance

The significance of NO and cytokinins in plant growth, development, and responses to abiotic stress is well-established.^2,43^ Although an early study by Romanov et al. (2008) suggested that NO does not directly activate the CK signal transduction and downstream components, subsequent research has provided insights into a more complex relationship between NO and CK signaling pathways.^40,81,86–90^ According to a study by Liu et al. (2013), CKs may act as an NO scavenger.^40^ In this work, the authors observed an *Arabidopsis* mutant line, *cnu* (*continuous NO unstressed*) with less sensitivity to NO.^40^ It was shown that *CNU1* is identical to the previously characterized *AMP1* (*altered meristem program 1*) gene.^40^ *AMP1* might encode a putative glutamate carboxypeptidase with an unknown biological role. In *amp1* mutants, a higher level of CKs was observed due to an increased CK biosynthesis mediated by an unknown mechanism.^40^ To test if the elevated CK content is responsible for the reduced sensitivity to NO in *cnu1*, the NO overproducing line *nox1* was treated with *trans*-zeatin. It was shown that almost all phenotypes of *nox1* such as NO-induced inhibition of seedling growth or reduced chlorophyll content in leaves were rescued by exogenously applied zeatin in a dose-dependent manner.^40^ A similar result was observed in the double mutant *cnu1–2 nox1*, which exhibits decreased NO levels compared to *nox1* and also a reduced severity of NO phenotype.^40^ The authors proposed that creatine kinase could potentially counteract the effects of NO through either functional antagonism or by chemically reducing endogenous NO levels.^40^ Additionally, their findings indicated that CKs and peroxynitrite could undergo reactions both *in vitro* and *in vivo*, resulting in the formation of various nitrated or nitrosylated CK forms, which might lose their biological activity.^40^ Consequently, CKs and NO could function as mutual suppressors, suggesting a dynamic regulatory interplay between these two signaling molecules.^40^

Feng et al. (2013) reported another interaction between CKs and NO.^81^ The NO-overproducing *Arabidopsis nox1* and *gsnor1–3 (GSNOR1)* mutant lines were defective in a number of cytokinin bioassays, such as cytokinin-mediated inhibition of root and hypocotyl elongation, cytokinin-mediated reduction of root cortex cells, and cytokinin-induced shoot regeneration from hypocotyl explants.^81^ Moreover, the expression of the primary CK response genes, the type-A response regulator genes were reduced, and the expression level and size of the expression domain of the synthetic *TCS-GFP* cytokinin reporter gene was reduced in *gsnor1-3*.^81^ Treating wildtype roots with NO released from GSNO and SNP yielded similar effects, implying that NO has an inhibitory effect on CK signaling.^81^ Further research showed that an *S*-nitrosylation occurs at Cys_115_ of the AHP1 protein.^81^ Cys_115_ is surrounded by Phe_103_ and Arg_114_, which build a typical acid-base/hydrophobic motif proposed for *S*-nitrosylation.^81^ A substitution of Cys_115_ with serine abolished the *S*-nitrosylation of AHP1 *in vitro* and *in vivo*, making CK signaling possible even in the presence of NO.^81^ Thus, *S*-nitrosylation of AHP1 represses its phosphorylation and inhibits the following transfer of the phosphate group to the ARRs.^81^ Therefore, the CK signaling is suppressed by NO at the level of the AHP proteins.^81^ *S*-nitrosylation has been observed in AHP2, AHP3, and AHP5 as well, further indicating the regulatory influence of NO on these components of the CK signaling pathway.^81^

Beside these direct interactions between NO and CK, there are more interactions between both molecules in an indirect manner. These indirect interactions can affect the development of tissues, the cell cycle, or responses to different stress conditions. For example, NO is a key signal molecule during flooding and hypoxic conditions.^2^ According to a study by Islam et al. (2022), CKs might also have beneficial effects during waterlogging.^91^ During both the duration of waterlogging and the following recovery period, it was shown that kinetin-sprayed waterlogged mungbean plants had significant improvements in shoot height, shoot dry weight, root dry weight and stem girth.^91^

A similar result was found by Huynh et al. (2005).^92^ Transgenic *Arabidopsis* plants carrying the *SAG12:ipt* (senescence associated gene 12) construct, which leads to increased CK biosynthesis in response to senescence, were used to delay the senescence induced by flooding stress.^92^ It was shown that after 3 and after 5 days of waterlogging transgenic plants accumulated more biomass than wild type plants.^92^ In a submergence experiment (3 d and 5 d), the transgenic plants recovered and grew significantly better than the wild type.^92^ Measurements of the CK content revealed a higher CK level for the transgenic plants in these experiments.^92^ For instance, in a recovery experiment following 3 d of submergence, *SAG12:ipt* plants had an amount of 700 ng∙g^−1^ CKs in dry weight, compared to 70 ng∙g^−1^ in wild type plants.^92^

Another NO-CKs interplay was observed by Lehotai et al. (2016).^87^ Selenite-exposed *Arabidopsis* revealed an increased NO content and a decreased CK content, and a mutually negative interplay between the two molecules was found in selenite-exposed roots.^87^ The study concluded that selenite modifies not only the CK metabolism and signal transduction, potentially through inhibition of root-to-shoot translocation and the regulation of CKX expression, but also the NO metabolism in an NR-independent process.^87^ Additionally, it has been suggested that there is an NO-CKs interaction, whereby both act as suppressor to each other’s signaling or level through a direct interaction in selenite-treated roots, resembling the notion of a mutually inhibitory interaction found by Liu et al. (2013) mentioned above.^87^ Interestingly, Lehotai et al. (2016) observed that the addition of benzyl adenine caused a reduction in NO content, even in roots not treated with selenite.^87^ In contrast, Tun et al. (2001) found that a hormone-depending increase in NO level occurs when cultured plant cells are treated with CKs.^89^ These apparently contradictory findings may be due to the different tissues and culture conditions used in both studies.

Shen et al. (2013) discovered an additional NO-CKs interaction, indicating that NO may act downstream of CK in the control of cell proliferation through CYCD3;1 (cyclin D-type protein).^88^ Using the NO-deficient line *nos1/noa1* of *Arabidopsis*, it was demonstrated that treatment with increasing levels of kinetin results in a reduced callus growth and greening compared to the wild type.^88^ The hypocotyl and root explants of *ahk4*, a CK receptor mutant impaired in CK-dependent callus induction, showed similar defects in green callus formation as *nos1/noa1*.^88^ Additionally, a slightly faster callus proliferation and greening phenotype was observed in the NO overproducer line *nox1–1* compared to the wild type.^88^ Exogenous treatment with benzyl adenine and treatment of wild type plants with the NO scavenger cPTIO led to the conclusion that NO acts as a second messenger, mediating the CK-induced transcriptional activation of C*YCD3;1* during cell proliferation, thus driving the mitotic cell cycle.^88^

Shen et al. (2013) observed an increase of the NO level when wild-type plants are treated with *trans*-zeatin.^88^ This finding is consistent with the finding of Tun et al. (2001), but not with Lehotai et al. (2016),^87–89^ probably due to different tissues and culture conditions.

Another type of crosstalk between NO and CKs was found by Yan, Shi and Gong (2021). In their work, they investigated the influence of GSNOR-mediated NO on the outgrowth of axillary buds of tomato (*Solanum lycopersicum* l.).^90^ Suppression of GSNOR, a key player involved in NO homeostasis, promoted apical and axillary bud outgrowth by inhibiting the expression of flavin monooxygenase (*FZY*).^90^ Together with an up-regulation of the auxin influx carriers (*AUXs*) and auxin efflux carriers (*PINs*) in axillary buds, there was a reduced accumulation of IAA (indoleacetic acid) in axillary buds.^90^ These alterations in IAA levels coupled with GSNOR-mediated changes in NO level increased CK biosynthesis, transport and signaling.^90^ The authors suggested that the increased polar auxin transporter stream (PATS) from axillary buds led to the accumulation of CKs, resulting in a significantly decreased IAA:CK ratio in axillary buds leading to the breaking of bud dormancy.^90^ This study reports that the interaction between NO and CK is realized indirectly by altering the homeostasis and signaling of auxin and CK in tomato plants.^90^

An interaction between NO and auxin occurs by nitrosylation of the auxin co-receptor TIR1 (TRANSPORT INHIBITOR RESPONSE 1).^93^ The auxin receptors TRANSPORT INHIBITOR RESPONSE 1/AUXIN SIGNALING F-BOX (TIR1/AFB) are F-box proteins that determine the substrate specificity of the Skp1-cullin 1-F-box (SCF) type E3 ubiquitin ligase complex.^94,95^ Binding of IAA results in an increased interaction with the auxin/indole-3-acetic acid (Aux/IAA) co-receptors.^94^ This interaction of Aux/IAA and TIR1/AFB leads to the ubiquitination and degradation of Aux/IAA.^94^ Aux/IAA act as repressor of the auxin response factors (ARF).^94^ IAA-mediated degradation of Aux/IAA therefore promotes the ARF activity and results in a transcriptional reprogramming.^94^ Apart from this, it has been shown that TIR1/AFB also contain an adenylate cyclase domain and its activity is increased by binding of IAA to TIR1/AFB.^94,96^ The following increased cAMP (cyclic adenosine monophosphate) level is also crucial for the final transcription response.^94,96^

Terrile et al. (2011) demonstrated that NO, released from NO donors, upregulates auxin-dependent gene expression, whereas Aux/IAA protein degradation is suppressed by the removal of NO.^93^ Furthermore, it has been shown that a *S*-nitrosylation at Cys_140_ enhances the TIR1 function and TIR1-Aux/IAA interaction, leading to Aux/IAA degradation, and ultimately promoting gene expression.^93^

Furthermore, it is shown that IAA-treated roots show an increased NO level.^24,93^ This interaction between NO and auxin is important since auxins and CKs act antagonistically.^41^ We therefore speculate that NO-mediated degradation of Aux/IAA proteins through TIR1 might affect the ratio of IAA:CK signaling. Additionally, a crosstalk between the NR and auxin in *Arabidopsis* has been reported.^97^ Using the *nia1 nia2* and *chl1–5* mutant lines, exhibiting a lower or higher NR activity compared to the wild type, respectively, it is demonstrated that low nitrogen conditions increased the auxin level, while high nitrogen decreased the auxin accumulation, compared to normal nitrogen conditions.^97^ In *chl1–5*, the auxin level was higher while the *nia1 nia2* double mutant showed a very low auxin level.^97^ The authors suggest that the NR activity is positively correlated with the root auxin level.^97^ The interaction between NO, root CK level, and auxin reveals a complex regulatory network influencing plant growth and development, where NO-mediated degradation of Aux/IAA proteins and NR activity play pivotal roles in modulating the balance between auxin and cytokinin signaling pathways. These findings highlight the interconnectedness of nitrogen metabolism, nitric oxide signaling, and hormone regulation in shaping plant physiological responses.

NO is also involved in plant immunity.^98^ Plants recognize pathogens through the perception of pathogen-associated molecular patterns (PAMP), resulting in the activation of PAMP-triggered immunity (PTI).^98,99^ Some pathogens can evade this basal immunity by blocking the defense response with effectors.^98,99^ Therefore, plants evolved genes for recognizing microbe effectors. By recognition of these effectors, the effector-triggered immunity (ETI) is induced.^98,99^ Ultimately, both PTI and ETI lead to the hypersensitive response (HR), in which programmed cell death of infected plant tissues is initiated.^98,99^ It was shown that NO plays a role in plant immunity during PTI, ETI and HR, and that the amounts of *S*-nitrosylated compounds are crucial for the defense responses.^98^ In addition, *Arabidopsis* mutants with increased NO levels caused by a mutation in *PGB1*, showed increased levels of jasmonic acid (JA) and ethylene, resulting in an increased resistance to *Botrytis cinerea*.^98^ It is noteworthy that the *S*-nitrosylation of SABP3 also takes place in the immune response of plants and that NO activates the salicylic acid (SA)-regulated defense pathway.^4,12,30,98^ CKs play a role in plant immunity, with increased levels enhancing resistance to infection and influencing ETI.^99^ NO modulates CK signaling by *S*-nitrosylation of AHP1, and CKs can potentially scavenge NO, leading to nitrosylated CKs with altered physiological activity.^40,81^ Considering NO’s impact on TIR1-Aux/IAA interaction and heightened sensitivity to auxin, along with the interplay between NO, CKs, and auxins, it is conceivable that these factors collaboratively fine-tune plant immune responses.

**Figure 3.**
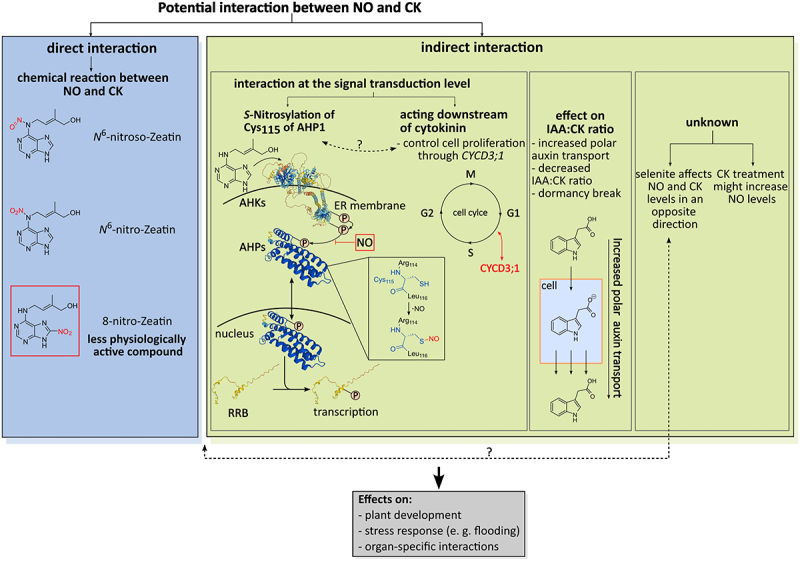
Known interactions between NO and CKs. Direct interaction: a potential NO and CK interaction occurs through a chemical reaction between both molecules, or between CKs and peroxynitrite as an intermediate product of further NO reactions. The resulting products exhibit different physiological activities. While 8-nitro-zeatin is physiologically less effective, *N^6^*-nitro and *N^6^*-nitroso-zeatin are approximately as effective as zeatin itself.^40^ Indirect interaction: the indirect interaction can be classified in interaction at the signal transduction level, affecting the IAA:CK ratio (IAA: indoleacetic acid), and in an interaction with an unknown intermediate player between CKs and NO. An interaction affecting the signal transduction was observed in the CK signal transduction (*S*-nitrosylation of AHP1).^81^ The signal transduction starts with binding of CKs to their receptor (AHK proteins; structure: AHK1 from *Arabidopsis thaliana*) which triggers an autophosphorylation within the AHK. The phosphate group (P) is subsequently transferred to the histidine containing phosphate transfer protein (AHP; structure: AHP1 from *Arabidopsis thaliana*). AHPs can shuttle between the cytosol and the nucleus and transfer the phosphate group to a conserved aspartate residue (D) of the response regulators (here response regulator type-B RRB, structure: RRB1 from *Vitis vinifera*). Phosphorylated RRB are active transcription factors. It was shown that an *S*-nitrosylation at Cys_115_ of AHP1 results in the inhibition of the CK signal transduction.^81^ The CK signal transduction scheme was modified after Kieber and Schaller (2014).^43^ The position of the phosphate group at the proteins does not show the actual position in the proteins and was set for a schematic overview. Three dimensional protein structures were obtained from AlphaFold.^10,11^ Another NO-CK interaction was reported by Shen et al. (2013), in which NO may function as a modulator in the CK-induced activation of *CYCD3;1* (cyclin D-type protein) during cell proliferation at G1-S cell cycle phase transition.^88^ This interaction may be similar to *S*-nitrosylation of AHP1 since here CK downstream signaling is suppressed. Yan, Shi and Gong (2021) demonstrated that NO affects the IAA:CK ratio through an increased polar auxin transport.^90^ This alteration of the IAA:CK ratio results in dormancy break in axillary buds.^90^ Schematic auxin transport modified after Armengot, Marques-Bueno and Jillais (2016).^100^ It was shown by Lehotai et al. (2016) that selenite oppositely affects the NO and CK metabolism.^87^ The authors suggest that both could act as suppresser of each other’s signaling/level, which may be a similar interaction found by Liu et al. (2013) and Feng et al. (2013).^87^ While Shen et al. (2013) and Tun et al. (2001) found that CKs increase the NO level, Lehotai et al. (2016) mentioned a decreased NO level after treatment with CKs.^87–89^ These finding suggest that there may be an interaction between NO and CKs mediated by an unknown player, which has not been determined yet. Probably, the reduced NO level reported by Lehotai et al. (2016) could be the same interaction between NO and CKs, which Liu et al. (2013) demonstrated. Dashed lines within this graphic represent possible connections between the found interactions of NO and CKs. Further abbreviations: cell cycle M: mitosis phase, G1: gap 1 phase, S: synthesis phase, G2: gap 2 phase.

Figure 3 summarizes known interactions between NO and CKs.

Additionally, it has been suggested that GSNOR-mediated NO could serve as a downstream signal of CK.^90^ This observation aligns with findings reported by Shen et al. (2013), who concluded that NO might function as a crucial second messenger for cytokinins in the processes of cell proliferation and re-differentiation during callus formation and shoot regeneration. Specifically, this involves the activation of CYCD3;1.^88^ For example, it is shown that both molecules affect the outgrowth of axillary buds and mediate responses to selenite stress providing evidence that NO and CKs play a regulatory role in abiotic stress signaling.^87,90^ Better understanding of the interaction between NO and CKs may lead to findings enabling mitigating strategies in breeding approaches leading to cultivars that are more tolerant toward waterlogging and hypoxia. Research has shown that NO mediates responses to hypoxic stress, which can occur through submergence.^2^ Under these circumstances, CKs have been found to have a beneficial effect.^92^ However, some evidence suggests that NO suppresses the beneficial effects of CKs. Hypoxia resulting from flooding or waterlogging, appears to induce or accelerate senescence, whereas CKs exhibit an anti-senescence effect by a promoting effect not only on cell division, but also on the chloroplast.^43,46,48,64,101–105^ Since flooding events are expected to become more frequent during the climate change,^106^ understanding the interaction between NO and CKs under hypoxic conditions such as waterlogging or flooding leading to both beneficial or detrimental effects is important to optimize breeding strategies to avoid yield loss.

## Open questions

Numerous questions remain unanswered concerning the interactions between NO and CKs, requiring more in-depth investigations: 1. Is it plausible for sugar conjugates of cytokinins to still function as NO scavengers? Theoretically, these conjugates may retain their scavenging ability, as nitrosylation typically does not occur at the position where sugars are bound. 2. How does the NO-CK crosstalk impact stress response and what are the critical implications for stress tolerance? This is particularly important as conflicting reports exist regarding the nature of the interplay between NO and CKs during stress situations, such as hypoxia. Some suggest a mutually inhibitory relationship, while others propose a (partial) positive feedback loop. 3. Could the *S*-nitrosylation of AHP1 and subsequent inhibition of CK signaling negatively impact hypoxia tolerance, given that CKs are known to exert beneficial effects under such circumstances? 4. Are there variations in the interaction between NO and CKs across different tissues? For instance, when roots experience waterlogging, does the interplay between NO and CKs differ from that observed in leaves? 5. Does the developmental stage of a plant play a role in influencing crosstalk between NO and CKs, particularly considering potential variations in gene expression? As plants progress through different developmental stages, the expression of genes involved in NO and CK signaling pathways may undergo dynamic changes. These alterations can potentially reshape the interaction between NO and CKs, leading to varying regulatory patterns and functional outcomes. Understanding how these molecular interactions evolve across the plant’s life cycle is crucial for unraveling the intricate regulatory networks that govern processes like growth, development, and stress responses.

Further research could contribute to answering these open questions.

1. The study of nitrosylated CK derivatives should include the conjugates of CK. Given the low nanomolar amounts of free CK bases in plant tissue, their effect on NO quenching is likely negligible. However, CK conjugates are present in much larger quantities as a storage form of CK and they might contribute significantly to the pool of NO scavenging molecules in plant tissues.^43,49^
2. The studies of Islam et al. (2022) and Huynh et al. (2005) demonstrated the beneficial effect of CKs during flooding stress. However, a connection to NO, a crucial signal molecule during flooding, is lacking. As NO scavenging is shown to be essential in preadapting plants to impending hypoxic conditions and since CK has shown a beneficial effects in response to waterlogging and submergence.^17,91,92^ Therefore, investigating the interplay between NO and CK is crucial for understanding plant adaptation to these circumstances. The interaction between NO and CKs during hypoxia should be investigated by utilizing mutant lines with altered NO and CK content in different combinations. Since CK conjugates might act as NO scavengers, mutant lines with increased levels of CK conjugates might have a reduced NO level, which might modulate preadaptation to hypoxia.^40^ In contrast, an increased CK status during hypoxia may prevent senescence, leading to a better performance during and after hypoxia.^91,92^ Quantification of NO and CKs followed under stress condition and their reassessment after the recovery period may offer additional insights into their potential interaction and its consequence on stress response. Furthermore, experiments involving mutants with NO and CK signal transduction components insensitive to modifications, such as *S*-nitrosylation, could be implemented in flooding and hypoxia studies. (3) The inhibition of CK signaling by *S*-nitrosylation of AHP1 was shown by Feng et al. (2013),^81^ which further suggest the importance of NO and CK crosstalk.

In summary, NO and CKs interact with each other in different manners. These interactions can be either direct through a chemical reaction or in an indirect manner, such as inhibiting CK signaling or affecting the auxin to CK ratio.^40,81,90^ As both molecules act as mediators in various plant functions such as stress mediators, the further research should not only focus on the potential outcome of their interaction on the stress response, but include also possible phycological and developmental effects. In this way, potential beneficial effects can be obtained and exploited against, for example flooding stress, which are expected to become more frequent due to the climate change.
